# Connections between body composition and dysregulation of islet α- and β-cells in type 2 diabetes

**DOI:** 10.1186/s13098-023-01250-3

**Published:** 2024-01-09

**Authors:** Jia-xi Miao, Jia-ping Xu, Rui Wang, Yu-xian Xu, Feng Xu, Chun-hua Wang, Chao Yu, Dong-mei Zhang, Jian-bin Su

**Affiliations:** 1 Department of Endocrinology, Affiliated Hospital 2 of Nantong University, and First People’s Hospital of Nantong, No.666 Shengli Road, Nantong, 226001 China; 2 Department of Clinical Laboratory, Affiliated Hospital 2 of Nantong University, and First People’s Hospital of Nantong, No.666 Shengli Road, Nantong, 226001 China; 3 Medical Research Center, Affiliated Hospital 2 of Nantong University, and First People’s Hospital of Nantong, No.666 Shengli Road, Nantong, 226001 China

**Keywords:** Body composition, C-peptide, Glucagon, Type 2 diabetes

## Abstract

**Background:**

Accompanying islet α- and β-cell dysregulation in type 2 diabetes (T2D) at the microscopic scale, alterations in body composition at the macroscopic scale may affect the pathogenesis of T2D. However, the connections between body composition and islet α-cell and β-cell functions in T2D have not been thoroughly explored.

**Methods:**

For this cross-sectional study, we recruited a total of 729 Chinese Han patients with T2D in a consecutive manner. Dual-energy X-ray absorptiometry (DXA) was used to measure body composition, which included total bone-free mass, total fat and lean mass, trunk fat and lean mass and limb fat and lean mass. Every patient underwent an oral glucose tolerance test to simultaneously detect glucose, C-peptide and glucagon. The indices of islet α-cell function included fasting glucagon levels and the area under the curve of glucagon after a challenge (AUC_glucagon_), while the indices of β-cell function included the insulin sensitivity index derived from C-peptide (ISI_C-peptide_) and the area under the curve of C-peptide after a challenge (AUC_C-peptide_).

**Results:**

Among all patients, fat mass, especially trunk fat mass, was significantly correlated with ISI_C-peptide_ and AUC_C-peptide_ levels (*r* = − 0.330 and 0.317, respectively,* p* < 0.001), while lean mass, especially limb lean mass, was significantly correlated with fasting glucagon and AUC_glucagon_ levels (*r* = − 0.196 and − 0.214, respectively,* p* < 0.001). Moreover, after adjusting for other relevant variables via multivariate linear regression analysis, increased trunk fat mass was independently associated with decreased ISI_C-peptide_ (*β* = − 0.247,* t* = − 3.628, *p* < 0.001, *partial R*^*2*^ = 10.9%) and increased AUC_C-peptide_ (*β* = 0.229,* t* = 3.581, *p* < 0.001, *partial R*^*2*^ = 8.2%), while decreased limb lean mass was independently associated with increased fasting glucagon (*β* = − 0.226,* t* = − 2.127, *p* = 0.034, *partial R*^*2*^ = 3.8%) and increased AUC_glucagon_ (*β* = − 0.218,* t* = − 2.050, *p* = 0.041, *partial R*^*2*^ = 2.3%). Additionally, when separate analyses were performed with the same concept for both sexes, we found that increased trunk fat mass was still independently associated with decreased ISI_C-peptide_ and increased AUC_C-peptide_, while decreased limb lean mass was still independently associated with increased fasting glucagon and AUC_glucagon_.

**Conclusions:**

Increased trunk fat mass may partly account for decreased insulin sensitivity and increased insulin secretion, while decreased limb lean mass may be connected to increased fasting glucagon and postprandial glucagon secretion.

**Supplementary Information:**

The online version contains supplementary material available at 10.1186/s13098-023-01250-3.

## Background

The resurging perspective on type 2 diabetes (T2D) reflects a bihormonal intricacy resulting in the dysregulation of glucagon secretion from islet α-cells and insulin secretion from β-cells [1, 2]. Islet α-cell dysfunction is characterized by glucagon secretion being reduced by half at low glucose concentrations and not being inhibited at high glucose concentrations, whereas islet β-cell dysfunction is characterized by insufficient compensatory insulin secretion to counteract increased insulin resistance [3, 4]. Extensive work is being undertaken worldwide to explore potential risk factors for bihormonal disorders, with the aim of developing innovative treatments that specifically focus on β-cells and insulin secretion as well as α-cells and glucagon secretion.

Accompanying islet α- and β-cell dysregulation in T2D at the microscopic scale, alterations in body composition may take part in the processes of T2D at the macroscopic scale. Body composition alterations in T2D patients are characterized by increases in whole-body fat mass, ectopic fat deposition (visceral fat accumulation), and a decrease in skeletal muscle mass [5, 6]. Is there any relationship between the macroscale of body composition and the microscale of α- and β-cell dysfunctions in the background of T2D? This question definitely warrants further investigation. In a previous study, there was an inverse correlation between the rate of annual increase in trunk fat and the change in β-cell function assessed by the glycaemic disposition index in young individuals who progressed to T2D [7]. Data from NHANES 1999 to 2004 (6147 men and 6369 women) revealed a positive correlation between DXA-measured metrics of adiposity (trunk fat mass, subtotal fat mass and total fat mass) and HOMA-IR (insulin resistance) and HOMA-β (β-cell function) [8]. A Korean study by Park et al. [5] revealed that lower skeletal muscle mass was associated with insulin resistance and diabetes. Anoop et al. [9] reported that elevated fasting plasma glucagon levels were positively associated with subcutaneous abdominal adipose tissue in 81 nonobese patients with T2D, but they failed to be associated with measures of lean muscle mass. Taken together, these findings strongly support the associations of body composition with measures of β-cell function. Nonetheless, studies on body composition and measures of α-cell function, especially postprandial glucagon measurements, are rare. Systematic study of the connections between body composition and the dysregulation of islet α-cells and β-cells in T2D patients is expected to be very worthwhile.

Hence, the present study was performed to investigate the relationship between body composition and the response of islet α- and β-cells in patients with T2D.

## Methods

### Study design and patient recruitment

The present study was part of a series we designed to explore potential risks for islet α- and β-cell dysfunctions. We posted a notice to recruit Chinese Han patients with T2D who visited the Endocrine and Metabolism Center of Affiliated Hospital 2 of Nantong University from October 2021 to July 2023. These patients came for outpatient appointments or were directed by community referrals. All eligible patients participated voluntarily. The research design involved evaluation and received approval from the Human Research Review Committee at this institution. The study flowchart is displayed in Additional file 1: Figure S1. During the recruitment stage, the inclusion criteria were as follows: (1) a diagnosis of T2D according to the reference published by the American Diabetes Association in 2021 [10]; (2) aged between 20 and 75 years; (3) underwent a DXA scan; and (4) demonstrated full understanding of the purpose and importance of this study and expressed willingness to participate. The exclusion criteria included the following: (1) existence of autoantibodies related to diabetes; (2) previous serious cardiovascular diseases, such as stroke, myocardial infarction, cardiovascular revascularization and peripheral arterial occlusion; (3) history of cancer; (4) presence of infectious illnesses; (5) chronic viral hepatitis or hepatic cirrhosis; (6) chronic kidney diseases, and estimated glomerular filtration rate (eGFR) < 60 mL/min/1.73m^2^; (7) received glucocorticoids or sex hormones; (8) connective tissue diseases and (9) past medical history of metabolic disorders or conditions impacting nutritional status, such as hyperthyroidism, hypothyroidism, Cushing syndrome. Finally, 729 patients with T2D with fully available data were included in this cross-sectional study. In accordance with the principles of the Declaration of Helsinki, the study design received approval from the ethical committee of Affiliated Hospital 2 of Nantong University, and all patients provided their consent by signing an informed consent form upon admission to the study.

### Data collection

Experienced and trained physicians gathered comprehensive clinical information from patients, encompassing demographic details (such as age, sex, weight, body mass index, and blood pressure), medical background (including diabetes duration, hypertension history, and smoking habits), prescription records (such as glucose-lowering medications and statin treatments), and biochemical measurements. The calculation of body mass index (BMI) involved dividing weight by the square of height (kg/m^2^). An automatic blood pressure monitor was used to measure blood pressure following a minimum of 30 min of rest. The details of patients’ glucose-lowering therapy were retrieved from the medical electronic record system, with categorization into several subgroups, such as drug naive, insulin treatments (variable assignment: no = 0, yes = 1), insulin secretagogues (no = 0, yes = 1), metformin (no = 0, yes = 1), thiazolidinediones (TZDs) (no = 0, yes = 1), α-glucosidase inhibitors (AGIs) (no = 0, yes = 1), dipeptidyl peptidase-4 inhibitors (DPP-4Is) (no = 0, yes = 1), sodium-glucose cotransporter-2 inhibitors (SGLT-2Is) (no = 0, yes = 1) and glucagon-like peptide-1 receptor agonists (GLP-1RAs) (no = 0, yes = 1).

After an 8-h fasting period, blood samples were obtained from peripheral veins to measure alanine aminotransferase (ALT), aspartate aminotransferase (AST), total bilirubin (TBI), high-density lipoprotein cholesterol (HDLC), low-density lipoprotein cholesterol (LDLC), and triglyceride (TG) levels. The estimated glomerular filtration rate (eGFR) was determined using the equation derived from the Modification of Diet in Renal Disease study [11].

### Assessment of pancreatic α- and β-cell functions

To evaluate the functions of pancreatic α- and β-cells, a 75-g oral glucose tolerance test (OGTT) was performed on all patients in the morning after fasting. All patients were advised to suspend all glucose-lowering therapies one day before the OGTT. Blood samples were collected from veins at 0, 30, 60, 120, and 180 min to simultaneously measure the levels of glucose in the serum, C-peptide in the serum, and glucagon in the plasma. To prevent interference with exogenous insulin, we utilized C-peptide as a substitute for insulin to assess β-cell function. Pancreatic islet β-cell function was assessed using the C-peptide-substituted Matsuda index (ISI_C-peptide_) and C-peptide area under the curve (AUC_C-peptide_), which serve as measures of insulin sensitivity and insulin secretion, respectively [12–14]. Pancreatic α-cell function was assessed using fasting glucagon and postchallenge glucagon levels, which were determined by calculating the area under the curve (AUC_glucagon_) during the OGTT. In addition, glucagon suppression at 30, 60, and 120 min during the OGTT was also assessed. Glucagon suppression at 30 min was glucagon_30min_/glucagon_0min_ (Glucagon_30min/0min_), glucagon suppression at 60 min was Glucagon_60min/0min_, and glucagon suppression at 120 min was Glucagon_120min/0min_.

Plasma glucagon was detected by a chemiluminescence immunoassay (product code: 20–1010; Glucagon Kit, JINDE BIOTECH, Guangzhou, China) with an automatic chemiluminescence immunoassay analyser (HomoG100, JINDE BIOTECH, Guangzhou, China). The glucagon kit is based on a double monoclonal antibody sandwich assay, with a percent relative bias of detection precision within ± 10%, intra-assay coefficients of variation (CV) < 10% and interassay CV < 10%. Serum C-peptide was detected by electrochemiluminescence (Elecsys C-Peptide, Roche Diagnostics GmbH, Germany) in an immunoassay analyser (Cobas e801, Roche, Germany). The accuracy of C-peptide detection was assessed through intraday precision (repeatability) and interday precision (intermediate precision). In our laboratory, the CV of repeatability was < 2.9%, and the CV of intermediate precision was < 3.6%.

### Dual-energy X-ray absorptiometry (DXA) for detecting body composition

Every subject in this study underwent DXA scanning (Discovery Wi, serial no. 86856; Hologic, Inc., Bedford, MA, USA) to assess body composition. The test was carried out by professionals from the appropriate medical technology department. Body composition parameters included total bone-free mass, total fat and lean mass, trunk fat and lean mass and limb fat and lean mass. The total fat/lean ratio and the trunk fat/lean ratio were calculated. Total bone-free mass referred to the sum of total fat and lean mass. The definition of the appendicular skeletal muscle mass index (ASMI) is the ratio of limb skeletal muscle mass (in kilograms) to the square of height (in square metres).

### Statistical analysis

The statistical analysis was performed with SPSS for Windows (version 25.0), and a threshold p value < 0.05 indicated statistical significance.

First, the clinical data were pooled and presented for all patients and for two subgroups according to sex. The means and standard deviations were used for normally distributed quantitative data, medians and interquartile ranges were used for skew-distributed quantitative data, and frequencies and percentages were used for qualitative data. The skewed indices were further natural log-transformed (ln). To analyse the differences between the two subgroups of men and women, Student’s t test (t value), the Mann–Whitney U test (standard Z value) or the Chi-square test (*x*^2^ value) were performed as appropriate. The test statistics (*t/Z/x*^2^ values) and corresponding *p* values are also provided.

Second, Pearson’s correlation was used to assess univariate correlations between body composition and indicators of islet α- and β-cell functions.

Third, when we identified which body composition metrics had the maximal correlation with indicators of islet α-cell and β-cell functions after Pearson’s correlation, we further explored whether these metrics were independently associated with indicators of α-cell and β-cell functions by controlling for other clinical covariates via multivariate linear regression analyses. During the multivariate linear regression, collinearity analysis was also performed using the variance inflation factor (VIF).

Fourth, considering that the body compositions of women and men may differ considerably from each other, analyses were performed separately for both sexes with the same concept.

## Results

### Clinical characteristics of patients

Table 1 displays the clinical baseline characteristics of all 729 T2D patients and two subgroups stratified by sex. All patients were from the Chinese Han population. Compared with women with T2D (n = 315), men with T2D (n = 414) were characterized by greater bone-free mass, total lean mass, trunk lean mass, limb lean mass and ASMI as well as lower total fat mass, total fat/lean ratio, trunk fat mass, trunk fat/lean ratio, limb fat mass, limb fat/lean ratio, fasting glucagon, and AUC_glucagon_. However, lnISI_C-peptide_ and lnAUC_C-peptide_ were comparable between the two subgroups. Other clinical indices, including DBP, TBI, ALT, albumin and UA, were greater in men with T2D than in women with T2D, while age, TC and HDLC were lower in men with T2D than in women with T2D. However, the two subgroups had comparable BMI, SBP, diabetes duration, incidence of hypertension, statin use, AST, TG, LDL-C, and HbA1c. Comparisons of antidiabetic treatments showed that men with T2D tended to have a lower frequency of metformin use, while the use of insulin, secretagogues, TZDs, AGIs, DPP-4Is, SGLT-2Is and GLP-1RAs was comparable.

**Table 1:** Clinical features of all recruited patients with T2D

Variables	Total	Men	Women	Test statistic	p value
*n*	729	414	315		
Age (year)	55.5 ± 10.8	54.4 ± 10.7	56.9 ± 10.7	− 3.174	0.002
BMI (kg/m^2^)	25.70 ± 3.60	25.80 ± 3.28	25.56 ± 3.98	0.898	0.370
SBP (mmHg)	134.0 ± 15.2	134.1 ± 14.9	133.9 ± 15.7	0.211	0.833
DBP (mmHg)	81.2 ± 9.9	82.3 ± 9.7	79.7 ± 10.0	3.451	0.001
Diabetes duration (year)	6.0 (3.0− 10.0)	5.0 (2.0− 10.0)	7.0 (3.0− 10.0)	− 1.775	0.076
Antidiabetic treatments					
Drug naive, *n* (%)	128 (17.6)	79 (19.1)	49 (15.6)	1.537	0.215
Insulin, *n* (%)	262 (35.9)	145 (35.0)	117 (37.1)	0.349	0.555
Secretagogues, *n* (%)	232 (31.8)	124 (30.0)	108 (34.3)	1.549	0.213
Metformin, *n* (%)	340 (46.6)	178 (43.0)	162 (51.4)	5.112	0.024
TZDs, *n* (%)	82 (11.2)	50 (12.1)	32 (10.2)	0.660	0.417
AGIs, *n* (%)	113 (15.5)	61 (14.7)	52 (16.5)	0.430	0.512
DPP-4Is, *n* (%)	62 (8.5)	35 (8.5)	27 (8.6)	0.003	0.955
SGLT-2Is, *n* (%)	105 (14.4)	64 (15.5)	41 (13.0)	0.866	0.352
GLP-1RAs, *n* (%)	45 (6.2)	26 (6.3)	19 (6.0)	0.019	0.890
Hypertension, *n* (%)	352 (48.3)	205 (49.5)	147 (46.7)	0.582	0.446
Statins uses, *n* (%)	132 (18.1)	74 (17.9)	58 (18.4)	0.035	0.852
ALT (U/L)	20 (14− 33)	22 (15− 33)	19 (13− 32)	2.011	0.044
AST (U/L)	18 (14− 25)	18 (13− 25)	19 (15− 25)	− 0.941	0.346
TBI (μmol/L)	11.19 ± 4.83	11.87 ± 4.78	10.30 ± 4.76	4.405	< 0.001
Albumin (g/L)	38.7 ± 4.3	39.1 ± 4.6	38.2 ± 3.9	2.741	0.006
TG (mmol/L)	1.90 (1.17− 3.22)	1.87 (1.10− 3.44)	1.93 (1.25− 2.88)	− 0.667	0.505
TC (mmol/L)	4.41 ± 1.08	4.33 ± 1.08	4.51 ± 1.07	− 2.222	0.027
HDLC (mmol/L)	1.14 ± 0.27	1.09 ± 0.26	1.20 ± 0.26	− 5.516	< 0.001
LDLC (mmol/L)	2.82 ± 0.90	2.79 ± 0.91	2.87 ± 0.89	− 1.282	0.2000
UA (μmol/L)	311.6 ± 102.2	331.0 ± 104.5	286.1 ± 93.3	6.016	< 0.001
eGFR (mL/min/1.73m^2^)	122.4 ± 34.0	121.3 ± 33.3	123.9 ± 34.9	− 1.019	0.308
HbA1c (%)	8.37 ± 1.85	8.39 ± 1.88	8.33 ± 1.80	0.487	0.627
Bone-free mass (kg)	69.71 ± 12.61	74.03 ± 11.53	64.04 ± 11.70	11.521	< 0.001
Total fat mass (kg)	21.72 ± 6.39	20.36 ± 5.74	23.51 ± 6.75	− 6.781	< 0.001
Total lean mass (kg)	47.99 ± 9.29	53.67 ± 7.03	40.53 ± 6.08	26.478	< 0.001
Total fat/lean ratio	0.463 ± 0.144	0.377 ± 0.087	0.576 ± 0.124	− 25.453	< 0.001
Trunk fat mass (kg)	12.29 ± 3.75	11.82 ± 3.63	12.90 ± 3.84	− 3.862	< 0.001
Trunk lean mass (kg)	23.90 ± 4.49	26.47 ± 3.56	20.52 ± 3.16	23.438	< 0.001
Trunk fat/lean ratio	0.522 ± 0.157	0.444 ± 0.115	0.625 ± 0.144	− 18.862	< 0.001
Limb fat mass (kg)	8.17 ± 2.91	7.22 ± 2.26	9.43 ± 3.18	− 10.992	< 0.001
Limb lean mass (kg)	19.96 ± 4.61	22.83 ± 3.51	16.20 ± 2.85	27.380	< 0.001
Limb fat/lean ratio	0.430 ± 0.177	0.315 ± 0.080	0.581 ± 0.156	− 29.852	< 0.001
ASMI (kg/m^2^)	7.10 ± 1.17	7.71 ± 0.96	6.30 ± 0.93	19.835	< 0.001
ISI_C-peptide_	649 (417− 1056)	646 (421− 1045)	658 (396− 1082)	− 1.497	0.134
lnISI_C-peptide_	6.53 ± 0.69	6.52 ± 0.69	6.53 ± 0.70	− 0.173	0.863
AUC_C-peptide_ (ng/mL·h)	8.76 (6.09− 13.19)	8.38 (5.99− 12.70)	9.15 (6.20− 13.97)	− 1.497	0.957
lnAUC_C-peptide_ (ng/mL·h)	2.17 ± 0.61	2.14 ± 0.60	2.21 ± 0.63	− 1.502	0.133
Fasting Glucagon (pg/mL)	121.0 ± 49.3	114.7 ± 48.9	129.3 ± 48.7	− 3.998	< 0.001
AUC_glucagon_ (pg/mL·h)	482.9 ± 179.3	461.6 ± 169.5	510.9 ± 188.0	− 3.710	< 0.001

To analyse the differences between the two subgroups of men and women, Student’s t test (*t* value), the Mann–Whitney U test (standard *Z* value) or the Chi-square test (*x*^2^ value) were performed as appropriate

*T2D* type 2 diabetes, *SBP/DBP* systolic/diastolic blood pressure, *BMI* body mass index, *TZDs* thiazolidinediones, *AGIs* α-glucosidase inhibitors, *DPP-4Is* dipeptidyl peptidase-4 inhibitors, *SGLT-2Is* sodium-glucose cotransporter-2 inhibitors, *GLP-1RAs* glucagon-like peptide-1 receptor agonists, *ALT* alanine aminotransferase, *AST* aspartate aminotransferase, *TBI* total bilirubin, *TG* triglyceride, *TC* total cholesterol, *HDLC* high-density lipoprotein cholesterol, *LDLC* low-density lipoprotein cholesterol, *UA* uric acid, *HbA1c* glycosylated hemoglobin A1c, *eGFR* estimated glomerular filtration rate, *Bone-free mass* sum total fat and muscle mass, ASMI appendicular skeletal muscle index, *ISI*_*C-peptide*_ C-peptide-substituted Matsuda’s index, *lnISI*_*C-peptide*_ natural log-transformed ISI_C-peptide_, *AUC*_*C-peptide*_ C-peptide area under curve during OGTT, *lnAUC*_*C-peptide*_ natural log-transformed AUC_C-peptide_, *AUC*_*glucagon*_ glucagon area under the curve during OGTT

### Univariate correlations of body composition with islet α- and β-cell functions

Univariate correlation analysis demonstrated that total bone-free mass was positively correlated with AUC_C-peptide_ (*r* = 0.211,* p* < 0.001) and was negatively correlated with ISI_C-peptide_, fasting glucagon and AUC_glucagon_ (*r* = − 0.266, − 0.123 and − 0.146, respectively,* p* < 0.001) (Table 2).

**Table 2:** Pearson’s correlation of body composition with islet α- and β-cell functions in all patients with T2D

Variables		lnISI_C-peptide_	lnAUC_C-peptide_	Fasting Glucagon	AUC_glucagon_
Bone-free mass	*r*	− 0.266	0.211	− 0.123	− 0.146
	*p*	< 0.001	< 0.001	0.001	< 0.001
Total fat mass	*r*	− 0.297	0.303	0.020	0.003
	*p*	< 0.001	< 0.001	0.588	0.941
Total lean mass	*r*	− 0.157	0.079	− 0.181	− 0.200
	*p*	< 0.001	< 0.001	< 0.001	< 0.001
Total fat/lean ratio	*r*	− 0.183	0.248	0.132	0.136
	*p*	< 0.001	< 0.001	< 0.001	< 0.001
Trunk fat mass	*r*	− 0.330	0.317	0.006	0.005
	*p*	< 0.001	< 0.001	0.882	0.885
Trunk lean mass	*r*	− 0.177	0.086	− 0.158	− 0.176
	*p*	< 0.001	0.021	< 0.001	< 0.001
Trunk fat/lean ratio	*r*	− 0.231	0.282	0.104	0.113
	*p*	< 0.001	< 0.001	0.005	0.002
Limb fat mass	*r*	− 0.220	0.254	0.043	0.021
	*p*	< 0.001	< 0.001	0.243	0.571
Limb lean mass	*r*	− 0.139	0.078	− 0.196	− 0.214
	*p*	< 0.001	< 0.001	< 0.001	< 0.001
Limb fat/lean ratio	*r*	− 0.105	0.181	0.149	0.148
	*p*	0.005	< 0.001	< 0.001	< 0.001
ASMI	*r*	− 0.159	0.113	− 0.178	− 0.185
	*p*	< 0.001	< 0.001	< 0.001	< 0.001

*Bone-free mass* sum total fat and muscle mass, *ASMI* appendicular skeletal muscle index, *ISI*_*C-peptide*_ C-peptide-substituted Matsuda index, *lnISI*_*C-peptide*_ natural log-transformed ISI_C-peptide_, *AUC*_*C-peptide*_ C-peptide area under curve during OGTT, *lnAUC*_*C-peptide*_ natural log-transformed AUC_C-peptide_

**Fig. 1:**
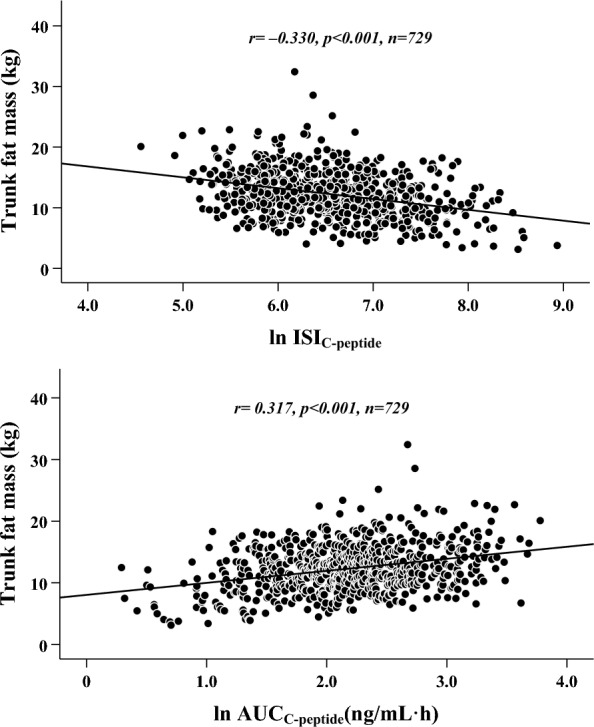
Graphical correlations between trunk fat mass and indicators of islet β-cell function in all patients with T2D

In terms of β-cell functions, all body composition metrics were positively correlated with the AUC_C-peptide_ and negatively correlated with the ISI_C-peptide_. Moreover, the correlations between fat mass metrics and β-cell functions were greater than those between lean mass metrics and β-cell functions. Furthermore, among the fat mass metrics, trunk fat mass was found to be most strongly correlated with ISI_C-peptide_ (*r* = − 0.330,* p* < 0.001) and AUC_C-peptide_ (*r* = 0.317,* p* < 0.001) (Table 2) (Fig. 1).

**Fig. 2:**
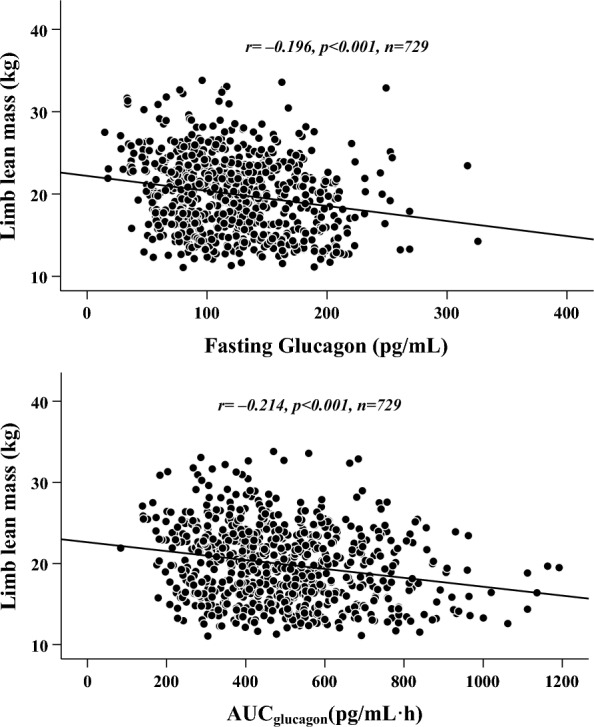
Graphical correlations between limb lean mass and indicators of islet α-cell function in all patients with T2D

In terms of α-cell functions, lean mass metrics were negatively correlated with fasting glucagon and AUC_glucagon_, but no significant relationships between fat mass metrics and α-cell functions were found. Furthermore, among the lean mass metrics, limb lean mass was found to be most strongly and negatively correlated with fasting glucagon (*r* = − 0.196,* p* < 0.001) and AUC_glucagon_ (*r* = − 0.214,* p* < 0.001) (Table 2) (Fig. 2).

### Independent effects of trunk fat mass on β-cell functions

When univariate correlations revealed that trunk fat mass was most strongly correlated with the ISI_C-peptide_ and AUC_C-peptide_ levels, we further used multivariate linear regression analyses to explore the independent effects of trunk fat mass on the ISI_C-peptide_ and AUC_C-peptide_ levels. After controlling for other clinical covariates, we found that trunk fat mass was negatively and independently associated with the ISI_C-peptide_ level (*β* = − 0.247, *t* = − 3.628, *p* < 0.001, partial *R*^*2*^ = 10.9%) and positively and independently associated with the AUC_C-peptide_ level (*β* = 0.229, *t* = 3.581, *p* < 0.001, partial* R*^*2*^ = 8.2%) (Table 3). Therefore, after adjustments for other clinical variables, trunk fat mass may independently explain 10.9% of the variation in the ISI_c-peptide_ and 8.2% of the variation in the AUC_c-peptide_ (Table 3).

**Table 3:** Impacts of body composition on outcomes of islet α- and β-cell functions according to multivariate linear regression analysis in all patients with T2D

Models	B (95% CI)	*β*	*t*	*p*	*Partial R*^*2*^
Impacts of trunk fat mass on lnISI_C-peptide_					
Model 0: crude	− 0.061 (− 0.074 to − 0.0148)	− 0.330	− 9.416	< 0.001	
Model 1: adjusted for age, sex, diabetes duration, BMI, SBP, DBP and statin medication	− 0.050 (− 0.073 to − 0.028)	− 0.274	− 4.449	< 0.001	
Model 2: additionally adjusted for ALT, albumin, lipid profiles, UA, eGFR and TBI	− 0.036 (− 0.058 to − 0.014)	− 0.196	− 3.213	0.001	
Model 3: additionally adjusted for HbA1c, fasting glucagon, AUC_glucagon_ and glucose-lowering therapies	− 0.037 (− 0.059 to − 0.016)	− 0.203	− 3.391	0.001	
Model 4: additionally adjusted for trunk lean mass, limb fat mass and limb lean mass	− 0.045 (− 0.070 to − 0.021)	− 0.247	− 3.628	< 0.001	10.9%
Impacts of trunk fat mass on lnAUC_C-peptide_					
Model 0: crude	0.052 (0.040 to 0.063)	0.317	9.014	< 0.001	
Model 1: adjusted for age, sex, diabetes duration, BMI, SBP, DBP a nd statin medication	0.041 (0.021 to 0.060)	0.252	4.117	< 0.001	
Model 2: additionally adjusted for ALT, albumin, lipid profiles, UA, eGFR and TBI	0.030 (0.011 to 0.050)	0.186	3.071	0.002	
Model 3: additionally adjusted for HbA1c, fasting glucagon, AUC_glucagon_ and glucose-lowering therapies	0.032 (0.014 to 0.050)	0.198	3.508	< 0.001	
Model 4: additionally adjusted for trunk lean mass, limb fat mass and limb lean mass	0.037 (0.017 to 0.058)	0.229	3.581	< 0.001	8.2%
Impacts of limb lean mass on fasting glucagon					
Model 0: crude	− 2.099 (− 2.862 to − 1.336)	− 0.196	− 5.402	< 0.001	
Model 1: adjusted for age, sex, diabetes duration, BMI, SBP, DBP and statin medication	− 2.322 (− 3.826 to − 0.817)	− 0.217	− 3.029	0.003	
Model 2: additionally adjusted for ALT, albumin, lipid profiles, UA, eGFR and TBI	− 2.787 (− 4.315 to − 1.259)	− 0.261	− 3.581	< 0.001	
Model 3: additionally adjusted for HbA1c, ISI_C-peptide_, AUC_C-peptide_ and glucose-lowering therapies	− 2.690 (− 4.244 to − 1.136)	− 0.251	− 3.399	0.001	
Model 4: additionally adjusted for trunk fat mass, trunk lean mass and limb fat mass	− 2.420 (− 4.654 to − 0.187)	− 0.226	− 2.127	0.034	3.8%
Impacts of limb lean mass on AUC_glucagon_					
Model 0: crude	− 8.295 (− 11.06 to − 5.532)	− 0.214	− 5.894	< 0.001	
Model 1: adjusted for age, sex, diabetes duration, BMI, SBP, DBP and statin medication	− 9.083 (− 14.49 to − 3.676)	− 0.234	− 3.298	0.001	
Model 2: additionally adjusted for ALT, albumin, lipid profiles, UA, eGFR and TBI	− 10.32 (− 15.85 to − 4.788)	− 0.266	− 3.664	< 0.001	
Model 3: additionally adjusted for HbA1c, ISI_C-peptide_, AUC_C-peptide_ and glucose-lowering therapies	− 8.920 (− 14.55 to − 3.293)	− 0.230	− 3.112	0.002	
Model 4: additionally adjusted for trunk fat mass, trunk lean mass and limb fat mass	− 8.454 (− 16.55 to − 0.357)	− 0.218	− 2.050	0.041	2.3%

*ISI*_*C-peptide*_ C-peptide-substituted Matsuda’s index, *lnISI*_*C-peptide*_ natural log-transformed ISI_C-peptide_, *AUC*_*C-peptide*_ C-peptide area under curve during OGTT, *lnAUC*_*C-peptide*_ natural log-transformed AUC_C-peptide_, *AUC*_*glucagon*_ glucagon area under the curve during OGTT, *BMI* body mass index, *ALT* alanine aminotransferase, *TBI* total bilirubin, *UA* uric acid, *HbA1c* glycosylated haemoglobin A1c, *eGFR* estimated glomerular filtration rate

### Independent effects of limb lean mass on α-cell functions

When univariate correlations revealed that limb lean mass was most strongly correlated with fasting glucagon and AUC_glucagon_ levels, we further used multivariate linear regression analyses to explore the independent effects of limb lean mass on fasting glucagon and AUC_glucagon_ levels. After controlling for other clinical covariates, we that limb lean mass was negatively and independently associated with ISI_C-peptide_ (*β* = − 0.226, *t* = − 2.127, *p* = 0.034, partial* R*^*2*^ = 3.8%) and AUC_C-peptide_ (*β* = − 0.218, *t* = − 2.050, *p* = 0.041, partial* R*^*2*^ = 2.3%) (Table 3). Therefore, after adjustments for other clinical variables, limb lean mass may independently explain 3.8% of the variation in fasting glucagon and 2.3% of the variation in AUC_glucagon_ (Table 3).

### Relationship between body composition and indicators of glucagon suppression

We also analysed the relationship between body composition and indicators of glucagon suppression in all patients with T2D (Additional file 2: Table S1; Additional file 3: Figure S2). Lean mass indices, especially limb lean mass, were significantly and negatively correlated with Glucagon_30min/0min_, Glucagon_60min/0min_ and Glucagon_120min/0min_ (*r* = − 0.090, − 0.127 and − 0.181, respectively; *p* < 0.05). However, we did not observe a clear correlation between measures of fat mass and indicators of glucagon suppression in these patients. After controlling for other clinical covariates by multivariate linear regression analysis, we found that limb lean mass was negatively and independently associated with Glucagon_120min/0min_ (*β* = − 0.155, *t* = − 2.070, *p* = 0.039, partial *R*^*2*^ = 3.3%). The impaired suppression of glucagon at 120 min exerted an important contribution to decreased limb lean mass.

### Separate analyses of the same concept for both sexes

Additionally, when separate analyses were performed with the same concept for both sexes, we found that increased trunk fat mass was still independently associated with decreased ISI_C-peptide_ and increased AUC_C-peptide_, while decreased limb lean mass was still independently associated with increased fasting glucagon and AUC_glucagon_. These results are shown in Additional file 4: Table S2; Additional file 5: Table S3; Additional file 6: Table S4; Additional file 7: Table S5.

## Discussion

In the present cross-sectional study in 729 patients with T2D, we explored the connections between body composition and dysregulations of islet α- and β-cells. Islet α-cell function was evaluated by fasting glucagon and AUC_glucagon_, and β-cell function was evaluated by ISI_C-peptide_ and AUC_C-peptide_. The primary findings of our research are as follows: first, the whole body mass has a link to islet α- and β-cell functions, involving a positive correlation of whole body mass with AUC_C-peptide_ and a negative correlation of whole body mass with fasting glucagon, AUC_glucagon_ and ISI_C-peptide_; second, after a subregional analysis, we found that the trunk fat mass was most prominently in a negative relationship with ISI_C-peptide_ and in a positive relationship with AUC_C-peptide_, while the limb lean mass was most prominently in a negative relationship with fasting glucagon and AUC_glucagon_; third, when separate analysis was performed with the same concept in both sexes, we found that increased trunk fat mass was still independently associated with decreased ISI_C-peptide_ and increased AUC_C-peptide_, while decreased limb lean mass was still independently associated with increased fasting glucagon and AUC_glucagon_. Therefore, there were close connections between macroscale body composition and microscale α- and β-cell dysfunctions in patients with T2D.

### Connections between body composition and β-cell dysfunction

In previous studies, fat mass indices were well known to be associated with measures of β-cell function [7, 8]. In our present study, we found that fat mass, especially trunk fat mass, was significantly correlated with decreased insulin sensitivity (ISI_C-peptide_) and increased insulin secretion (AUC_C-peptide_). Our results were in accordance with the previous literature. What, then, is the underlying mechanism? In adult populations, compelling evidence suggests that the link between excessive body fat and insulin resistance stems from the inability of adipose tissue to effectively store surplus energy intake [15]. Failed storage leads to the accumulation of fat in unintended places, disrupting important metabolic processes and, over time, causing damage to β-cells [16, 17]. Previous research has also demonstrated that among a cohort of youth at risk for diabetes, there is an independent and positive relationship between total body fat mass and insulin secretion; conversely, there is a negative association with insulin sensitivity [18, 19]. Given the strong correlation observed between abdominal obesity and the presence of ectopic fat deposits (fat stored in atypical locations), it is reasonable to surmise that an increase in abdominal fat can serve as a surrogate marker for heightened ectopic fat accumulation[7]. This finding lends support to the hypothesis that abdominal obesity may play an important role in β-cell dysfunction.

Conflicting results concerning the relationship between muscle mass indices and insulin sensitivity have been reported. Usually, greater muscle mass is associated with greater insulin sensitivity. Park et al.[5] reported that lower skeletal muscle mass is associated with greater insulin resistance. However, previous studies have shown that a greater muscle mass index is associated with greater insulin resistance. Sakai et al. [20] reported that there was a significant positive correlation between the appendicular muscle mass index (AMI) and clinical parameters of β-cell function (HOMA-β) and insulin resistance (HOMA-IR) in both men and women. Ma et al. [21] also reported that regional lean mass indices, including trunk, arm and leg mass indices, were positively correlated with HOMA-IR in both females and males. In our present study, we found that lean mass indices were positively correlated with AUC_C-peptide_ and inversely correlated with ISI_C-peptide_. Furthermore, in a recent study, Chen et al. [22] reported a U-shaped association between plasma C-peptide and sarcopenia in elderly Chinese patients with diabetes. Therefore, we speculate that the relationship between muscle mass and insulin sensitivity/insulin resistance may be bidirectional. However, further studies with larger sample sizes will be needed to confirm these findings.

### Connections between body composition and α-cell dysfunction

Few human studies have analysed body composition alterations and islet α-cell function, especially postprandial glucagon measurements. In a small sample of nonobese patients with T2D (n = 81), elevated fasting plasma glucagon levels were found to be positively associated with subcutaneous abdominal adipose tissue [9]. Although the associations did not reach statistical significance, primarily due to the small sample size (n = 81), fasting plasma glucagon levels were found to be negatively correlated with lean muscle mass (*r* =  − 0.10 ~  − 0.12, *p* = 0.30 ~ 0.37). Laurenti et al. [23] conducted a study to assess individual and standardized glucagon kinetics in healthy humans and found that the glucagon clearance rate was positively correlated with lean body mass (LBM). Decreased clearance of glucagon could contribute to increased glucagon levels, which in turn could lead to decreased lean body mass. Our present study systematically reported the connections between body composition and the dysregulation of islet α-cells in T2D patients in a large cohort. In our present study, lower lean mass, especially limb lean mass, was significantly correlated with higher fasting glucagon and AUC_glucagon_ levels (*r* = − 0.196 and − 0.214, respectively; p < 0.001). However, our study did not find relationships of fasting glucagon or AUC_glucagon_ with measurements of fat mass by DXA. The main reason may be that the patients in our study were treated with multiple glucose-lowering drugs, which had effects on body composition. Another possible reason was that 77.4% (n = 564) of participants had a relatively lower BMI (< 28 kg/m^2^).

In addition, we analysed the relationship between body composition and indicators of glucagon suppression (*i.e.,* Glucagon_30min/0min_, Glucagon_60min/0min_ and Glucagon_120min/0min_) in all patients with T2D. Limb lean mass was significantly and inversely correlated with Glucagon_120min/0min_ (*r* = − 0.181, p < 0.001). After controlling for other clinical covariates, limb lean mass was found to be negatively and independently associated with Glucagon_120min/0min_ (*β* = − 0.155, *t* = − 2.070, *p* = 0.039; partial *R*^*2*^ = 3.3%). The impaired suppression of glucagon at 120 min exerted an important contribution to decreased limb lean mass. Glucagon-like peptide-1 (GLP-1), a gut incretin hormone, may inhibit glucagon secretion directly from α-cells. In a previous study, Færch et al. [24] demonstrated that GLP-1 was not associated with early glucagon suppression, whereas positive changes in GLP-1 levels from 30 to 120 min during the OGTT were associated with greater late glucagon suppression. Therefore, clinical strategies with GLP-1 analogues targeting the inhibition of postchallenge glucagon may be beneficial for improving limb lean mass.

### Evidence for connections between decreased lean mass and α-cell dysfunction

There are several lines of evidence linking lean mass atrophy and α-cell dysfunction. Insulin is an anabolic hormone and may lead to body weight gain [25], whereas glucagon is a counter-regulatory hormone and may decrease body weight [26]. Chronic glucagon treatment can reduce lean body mass [27]. Among the experimental Sprague‒Dawley rats, those that received insulin treatment alone experienced an increase in both lean and fat mass. However, in the group of rats that received four times the amount of insulin plus glucagon, there was a significant increase in fat mass only. This indicated that the addition of glucagon prevented further increases in lean mass [28]. Ueno et al. [29] revealed that mice lacking proglucagon-derived peptides exhibited muscle fibre hypertrophy, suggesting that inhibiting glucagon activity resulted in enhanced skeletal muscle mass in these mice. Okun et al. [30] tested blood serum levels of glucagon in mouse models of obesity-related T2D, and mice with profound hyperglycaemia and skeletal muscle atrophy exhibited high glucagon levels. Mechanistically, liver alanine catabolism mediated by the activation of glucagon signalling promoted hyperglycaemia and skeletal muscle atrophy [30]. Taken together, these direct and indirect findings are supportive of islet α-cell dysfunction marked by elevated glucagon levels (hyperglucagonaemia) leading to a decrease in lean mass in patients with T2D.

## Limitations

A few limitations of our study need to be noted. First, this research is a cross-sectional investigation of the cause-and-effect connection between body composition and markers of pancreatic α-cell and β-cell functionalities. Consequently, additional longitudinal studies and fundamental experimental exploration are imperative for further understanding of this topic. Second, some glucose-lowering drugs, such as SGLT-2Is and GLP-1RAs, may have an effect on body composition. The ideal approach is to perform the study in T2D patients without the use of glucose-lowering drugs. However, in clinical practice, it is difficult to collect a large sample of T2D patients not using glucose-lowering drugs. Third, our study was limited to Chinese Han patients with T2D in a single centre, making the generalizability of the findings to other ethnic groups very questionable. Fourth, our study lacked a control group of healthy individuals, preventing us from determining the relationships between body composition and pancreatic α-cell and β-cell function in healthy controls. Fifth, we did not collect data on exercise, which may have an effect on insulin sensitivity or body composition. Sixth, considering menopause in women and the muscle-mass effect of declining testosterone levels in aging men, it might also be good to check the results in four subgroups (premenopausal woman, postmenopausal woman, young to middle-aged man, and elderly man). However, the sample size in the present study limited further grouping and analysis.

## Conclusions

In summary, increased trunk fat mass may partly account for decreased insulin sensitivity and increased insulin secretion, while decreased limb lean mass may be connected to increased fasting glucagon and postprandial glucagon secretion. Clinical strategies targeting improvements in islet α-cell and β-cell functions may be conducive to optimizing body composition.

### Supplementary Information

**Additional file 1: Figure S1.** Study flowchart.

**Additional file 2: Table S1.** Pearson’s correlation of body composition with indicators of glucagon suppression in all patients with T2D.

**Additional file 3: Figure S2.** Graphically displayed correlations between limb lean mass and indicators of glucagon suppression in all patients with T2D (Glucagon_30min/0min_: glucagon suppression at 30 min; Glucagon_60min/0min_: glucagon suppression at 60 min; Glucagon_120min/0min_: glucagon suppression at 120 min).

**Additional file 4: Table S2.** Pearson’s correlation of body composition with islet α-cell and β-cell functions in men with T2D (*n* = 414).

**Additional file 5: Table S3.** Pearson’s correlation of body composition with islet α- and β-cell functions in women with T2D (*n* = 315).

**Additional file 6: Table S4.** Impact of body composition on outcomes of islet α- and β-cell functions according to multivariate linear regression analysis in men with T2D (*n* = 414).

**Additional file 7: Table S5.** Impact of body composition on outcomes of islet α- and β-cell functions according to multivariate linear regression analysis in women with T2D (*n* = 315).

## Data Availability

The data for this study are available from the principal investigators upon reasonable request.

## Abbreviations

T2D: Type 2 diabetes
DXA: Dual X-ray absorptiometry
SBP/DBP: Systolic/diastolic blood pressure
BMI: Body mass index
TZDs: Thiazolidinediones
AGIs: α-Glucosidase inhibitors
DPP-4Is: Dipeptidyl peptidase-4 inhibitors
SGLT-2Is: Sodium-glucose cotransporter-2 inhibitors
GLP-1RAs: Glucagon-like peptide-1 receptor agonists
ALT: Alanine aminotransferase
AST: Aspartate aminotransferase
TBI: Total bilirubin
TG: Triglyceridestriglyceride
TC: Total cholesterol
HDLC: High-density lipoprotein cholesterol
LDLC: Low-density lipoprotein cholesterol
UA: Uric acid
HbA1c: Glycosylated hemoglobin A1c
eGFR: Estimated glomerular filtration rate
Bone-free mass: Sum total fat and muscle mass
ASMI: Appendicular skeletal muscle index
ISI_C__-peptide_: C-peptide-substituted Matsuda’s index
AUC_C__-peptide_: C-peptide area under curve during OGTT
AUC_glucagon_: Glucagon area under the curve during OGTT
