# Supplementary figures and images for: Connections between body composition and dysregulation of islet α- and β-cells in type 2 diabetes
Source: Diabetol Metab Syndr. 2024 Jan 9;16:11. doi: 10.1186/s13098-023-01250-3 (PMC10775650; doi:10.1186/s13098-023-01250-3)

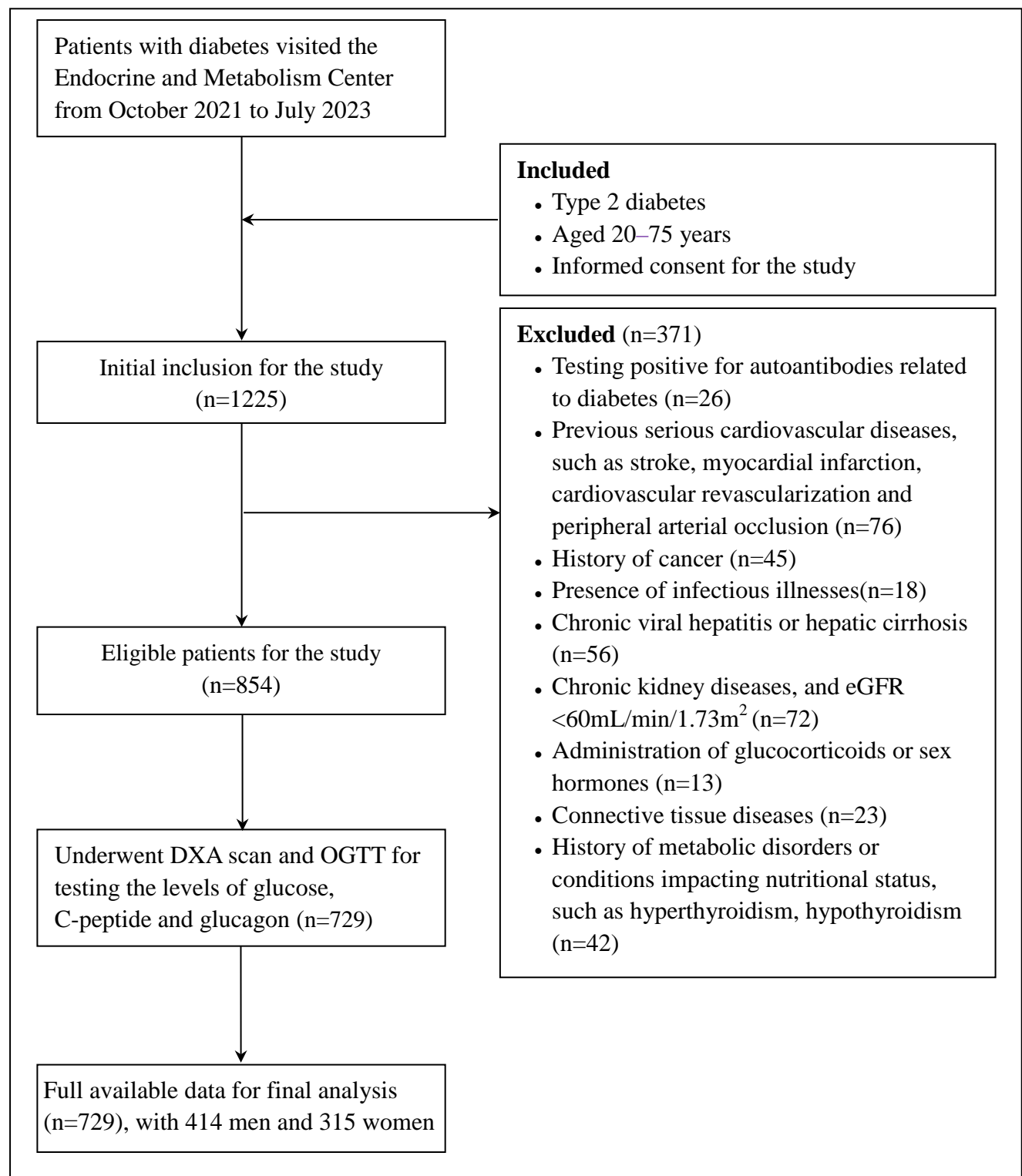

**Figure S1.** Study flowchart

Supplement: Supplementary file 1 — Additional file 1: Figure S1. Study flowchart. [file 13098_2023_1250_MOESM1_ESM.pdf]
