# Supplementary material for: Connections between body composition and dysregulation of islet α- and β-cells in type 2 diabetes
Source: Diabetol Metab Syndr. 2024 Jan 9;16:11. doi: 10.1186/s13098-023-01250-3 (PMC10775650; doi:10.1186/s13098-023-01250-3)
Supplement: Supplementary file 2 — Additional file 2: Table S1. Pearson’s correlation of body composition with indicators of glucagon suppression in all patients with T2D. [file 13098_2023_1250_MOESM2_ESM.docx]

**Table S1** Pearson’s correlation of body composition with indicators of glucagon suppression in all patients with T2D

| **Variables** | | **Glucagon_30min/0min_** | **Glucagon_60min/0min_** | **Glucagon_120min/0min_** |
| --- | --- | --- | --- | --- |
| **Bone-free mass** | ***r*** | –0.092 | –0.127 | –0.176 |
|  | ***p*** | 0.013 | 0.001 | <0.001 |
| **Total fat mass** | ***r*** | –0.047 | –0.067 | –0.065 |
|  | ***p*** | 0.199 | 0.069 | 0.080 |
| **Total lean mass** | ***r*** | –0.088 | –0.091 | –0.136 |
|  | ***p*** | 0.017 | 0.013 | <0.001 |
| **Total fat/lean ratio** | ***r*** | 0.016 | 0.021 | 0.059 |
|  | ***p*** | 0.660 | 0.572 | 0.109 |
| **Trunk fat mass** | ***r*** | –0.041 | –0.064 | –0.066 |
|  | ***p*** | 0.264 | 0.084 | 0.075 |
| **Trunk lean mass** | ***r*** | –0.085 | –0.082 | –0.132 |
|  | ***p*** | 0.021 | 0.026 | <0.001 |
| **Trunk fat/lean ratio** | ***r*** | 0.016 | 0.008 | 0.042 |
|  | ***p*** | 0.670 | 0.824 | 0.251 |
| **Limb fat mass** | ***r*** | –0.043 | –0.057 | –0.049 |
|  | ***p*** | 0.243 | 0.122 | 0.183 |
| **Limb lean mass** | ***r*** | –0.090 | –0.127 | –0.181 |
|  | ***p*** | 0.014 | 0.001 | <0.001 |
| **Limb fat/lean ratio** | ***r*** | 0.016 | 0.032 | 0.072 |
|  | ***p*** | 0.669 | 0.381 | 0.051 |
| **ASMI** | ***r*** | –0.069 | –0.094 | –0.148 |
|  | ***p*** | 0.063 | 0.011 | <0.001 |

Bone-free mass: sum total fat and muscle mass; ASMI: appendicular skeletal muscle index; Glucagon_30min/0min_: glucagon suppression at 30min; Glucagon_60min/0min_: glucagon suppression at 60min; Glucagon_120min/0min_: glucagon suppression at 120min.
