# Supplementary material for: Connections between body composition and dysregulation of islet α- and β-cells in type 2 diabetes
Source: Diabetol Metab Syndr. 2024 Jan 9;16:11. doi: 10.1186/s13098-023-01250-3 (PMC10775650; doi:10.1186/s13098-023-01250-3)
Supplement: Supplementary file 3 — Additional file 3: Figure S2. Graphically displayed correlations between limb lean mass and indicators of glucagon suppression in all patients with T2D (Glucagon30min/0min: glucagon suppression at 30 min; Glucagon60min/0min: glucagon suppression at 60 min; Glucagon120min/0min: glucagon suppression at 120 min). [file 13098_2023_1250_MOESM3_ESM.pdf]

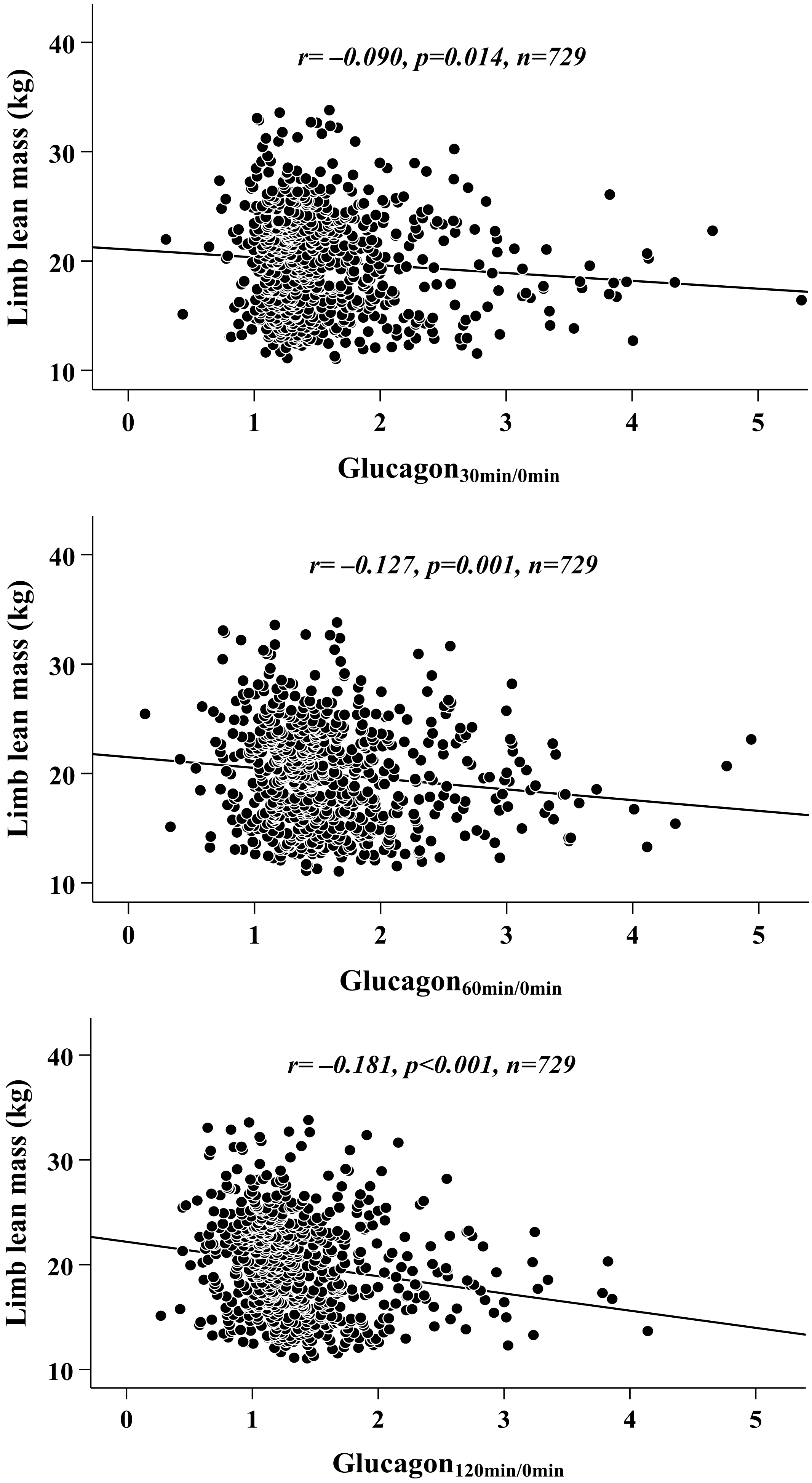

Figure S2. Graphically exhibited correlations between limb lean mass and indicators of glucagon suppression in all patients with T2D (Glucagon suppression at 30min: $Glucagon_{30min/0min}$; glucagon suppression at 60min: $Glucagon_{60min/0min}$; glucagon suppression at 120min: $Glucagon_{120min/0min}$).
