# Supplementary material for: Connections between body composition and dysregulation of islet α- and β-cells in type 2 diabetes
Source: Diabetol Metab Syndr. 2024 Jan 9;16:11. doi: 10.1186/s13098-023-01250-3 (PMC10775650; doi:10.1186/s13098-023-01250-3)
Supplement: Supplementary file 4 — Additional file 4: Table S2. Pearson’s correlation of body composition with islet α-cell and β-cell functions in men with T2D (n = 414). [file 13098_2023_1250_MOESM4_ESM.docx]

**Table S2** Pearson’s correlation of body composition with islet α-cell and β-cell functions in men with T2D (*n*=414)

| **Variables** | | **lnISI_C-peptide_** | **lnAUC_C-peptide_** | **Fasting Glucagon** | **AUC_glucagon_** |
| --- | --- | --- | --- | --- | --- |
| **Bone-free mass** | ***r*** | –0.344 | 0.300 | –0.083 | –0.112 |
|  | ***p*** | <0.001 | <0.001 | 0.090 | 0.023 |
| **Total fat mass** | ***r*** | –0.385 | 0.341 | 0.016 | 0.046 |
|  | ***p*** | <0.001 | <0.001 | 0.747 | 0.350 |
| **Total lean mass** | ***r*** | –0.250 | 0.214 | –0.124 | –0.145 |
|  | ***p*** | <0.001 | <0.001 | 0.012 | 0.003 |
| **Total fat/lean ratio** | ***r*** | –0.348 | 0.314 | 0.048 | 0.033 |
|  | ***p*** | <0.001 | <0.001 | 0.332 | 0.497 |
| **Trunk fat mass** | ***r*** | **–0.407** | **0.346** | 0.012 | 0.028 |
|  | ***p*** | **<0.001** | **<0.001** | 0.811 | 0.565 |
| **Trunk lean mass** | ***r*** | –0.273 | 0.205 | –0.094 | –0.101 |
|  | ***p*** | <0.001 | <0.001 | 0.057 | 0.040 |
| **Trunk fat/lean ratio** | ***r*** | –0.364 | 0.327 | 0.032 | 0.025 |
|  | ***p*** | <0.001 | <0.001 | 0.522 | 0.611 |
| **Limb fat mass** | ***r*** | –0.320 | 0.307 | 0.016 | 0.063 |
|  | ***p*** | <0.001 | <0.001 | 0.740 | 0.201 |
| **Limb lean mass** | ***r*** | –0.223 | 0.218 | **–0.144** | **–0.174** |
|  | ***p*** | <0.001 | <0.001 | **0.003** | **<0.001** |
| **Limb fat/lean ratio** | ***r*** | –0.269 | 0.252 | 0.060 | 0.034 |
|  | ***p*** | <0.001 | <0.001 | 0.223 | 0.490 |
| **ASMI** | ***r*** | –0.221 | 0.223 | 0.111 | 0.136 |
|  | ***p*** | <0.001 | <0.001 | 0.024 | 0.006 |

Bone-free mass: sum total fat and muscle mass; ASMI: appendicular skeletal muscle index; ISI_C-peptide_: C-peptide-substituted Matsuda’s index; lnISI_C-peptide_: natural log-transformed ISI_C-peptide_; AUC_C-peptide_: C-peptide area under curve during OGTT; lnAUC_C-peptide_: natural log-transformed AUC_C-peptide_
