# Supplementary material for: Connections between body composition and dysregulation of islet α- and β-cells in type 2 diabetes
Source: Diabetol Metab Syndr. 2024 Jan 9;16:11. doi: 10.1186/s13098-023-01250-3 (PMC10775650; doi:10.1186/s13098-023-01250-3)
Supplement: Supplementary file 5 — Additional file 5: Table S3. Pearson’s correlation of body composition with islet α- and β-cell functions in women with T2D (n = 315). [file 13098_2023_1250_MOESM5_ESM.docx]

**Table S3** Pearson’s correlation of body composition with islet α- and β-cell functions in women with T2D (*n*=315)

| **Variables** | | **lnISI_C-peptide_** | **lnAUC_C-peptide_** | **Fasting Glucagon** | **AUC_glucagon_** |
| --- | --- | --- | --- | --- | --- |
| **Bone-free mass** | ***r*** | –0.215 | 0.198 | –0.081 | –0.089 |
|  | ***p*** | <0.001 | <0.001 | 0.151 | 0.113 |
| **Total fat mass** | ***r*** | –0.226 | 0.255 | 0.039 | 0.017 |
|  | ***p*** | <0.001 | <0.001 | 0.490 | 0.758 |
| **Total lean mass** | ***r*** | –0.112 | 0.097 | –0.113 | –0.153 |
|  | ***p*** | 0.004 | 0.086 | 0.046 | 0.007 |
| **Total fat/lean ratio** | ***r*** | –0.184 | 0.272 | 0.027 | 0.082 |
|  | ***p*** | <0.001 | <0.001 | 0.637 | 0.148 |
| **Trunk fat mass** | ***r*** | **–0.247** | **0.274** | 0.042 | 0.022 |
|  | ***p*** | **<0.001** | **<0.001** | 0.462 | 0.701 |
| **Trunk lean mass** | ***r*** | –0.168 | 0.102 | –0.087 | –0.137 |
|  | ***p*** | 0.003 | 0.071 | 0.123 | 0.015 |
| **Trunk fat/lean ratio** | ***r*** | –0.212 | 0.268 | 0.004 | 0.059 |
|  | ***p*** | <0.001 | <0.001 | 0.937 | 0.294 |
| **Limb fat mass** | ***r*** | –0.173 | 0.207 | 0.031 | 0.009 |
|  | ***p*** | 0.002 | <0.001 | 0.584 | 0.880 |
| **Limb lean mass** | ***r*** | –0.145 | 0.094 | **–0.136** | **–0.162** |
|  | ***p*** | 0.011 | 0095 | **0.018** | **0.004** |
| **Limb fat/lean ratio** | ***r*** | –0.105 | 0.195 | 0.056 | 0.097 |
|  | ***p*** | 0.057 | <0.001 | 0.328 | 0.086 |
| **ASMI** | ***r*** | –0.156 | 0.115 | –0.133 | –0.124 |
|  | ***p*** | 0.006 | 0.041 | 0.018 | 0.028 |

Bone-free mass: sum total fat and muscle mass; ASMI: appendicular skeletal muscle index; ISI_C-peptide_: C-peptide-substituted Matsuda’s index; lnISI_C-peptide_: natural log-transformed ISI_C-peptide_; AUC_C-peptide_: C-peptide area under curve during OGTT; lnAUC_C-peptide_: natural log-transformed AUC_C-peptide_
