# Supplementary material for: Connections between body composition and dysregulation of islet α- and β-cells in type 2 diabetes
Source: Diabetol Metab Syndr. 2024 Jan 9;16:11. doi: 10.1186/s13098-023-01250-3 (PMC10775650; doi:10.1186/s13098-023-01250-3)
Supplement: Supplementary file 6 — Additional file 6: Table S4. Impact of body composition on outcomes of islet α- and β-cell functions according to multivariate linear regression analysis in men with T2D (n = 414). [file 13098_2023_1250_MOESM6_ESM.docx]

**Table S4** Effects of body composition on outcomes of islet α- and β-cell functions according to multivariate linear regression analysis in men with T2D (n=414)

| **Models** | **B (95% CI)** | ***β*** | ***t*** | ***p*** | ***Partial R^2^*** |
| --- | --- | --- | --- | --- | --- |
| **Impacts of trunk fat mass on lnISI_C-peptide_** |  |  |  |  |  |
| Model 0: crude; | –0.077(–0.094 to –0.060) | –0.407 | –9.019 | <0.001 |  |
| Model 1: adjusted for age, diabetes duration, BMI, SBP, DBP and statin medication; | –0.055(–0.084 to –0.027) | –0.292 | –3.845 | <0.001 |  |
| Model 2: additionally adjusted for ALT, albumin, lipid profiles, UA, eGFR and TBI; | –0.039(–0.068 to –0.011) | –0.207 | –2.711 | 0.007 |  |
| Model 3: additionally adjusted for HbA1c, fasting glucagon, AUC_glucagon_ and glucose-lowering therapies | –0.039(–0.068 to –0.011) | –0.208 | –2.759 | 0.006 |  |
| Model 4: additionally adjusted for trunk lean mass and limb lean mass | –0.038(–0.066 to –0.009) | –0.198 | –2.622 | 0.009 | 16.6% |
| **Impacts of trunk fat mass on lnAUC_C-peptide_** |  |  |  |  |  |
| Model 0: crude; | 0.057(0.042 to 0.072) | 0.346 | 7.479 | <0.001 |  |
| Model 1: adjusted for age, diabetes duration, BMI, SBP, DBP and statin medication; | 0.039(0.014 to 0.064) | 0.236 | 3.076 | 0.002 |  |
| Model 2: additionally adjusted for ALT, albumin, lipid profiles, UA, eGFR and TBI; | 0.026 (0.001 to 0.051) | 0.158 | 2.056 | 0.040 |  |
| Model 3: additionally adjusted for HbA1c, fasting glucagon, AUC_glucagon_ and glucose-lowering therapies | 0.029(0.005 to 0.052) | 0.174 | 2.413 | 0.016 |  |
| Model 4: additionally adjusted for trunk lean mass and limb lean mass | 0.028(0.005 to 0.051) | 0.170 | 2.310 | 0.019 | 10.4% |
| **Impacts of limb lean mass on fasting glucagon** |  |  |  |  |  |
| Model 0: crude; | –2.006(–3.343 to –0.670) | –0.144 | –2.950 | 0.003 |  |
| Model 1: adjusted for age, diabetes duration, BMI, SBP, DBP and statin medication; | –2.484(–4.391 to –0.577) | –0.178 | –2.561 | 0.011 |  |
| Model 2: additionally adjusted for ALT, albumin, lipid profiles, UA, eGFR and TBI; | –2.597(–4.549 to –0.646) | –0.186 | –2.617 | 0.009 |  |
| Model 3: additionally adjusted for HbA1c, ISI_C-peptide_, AUC_C-peptide_ and glucose-lowering therapies; | –2.408(–4.409 to –0.408) | –0.172 | –2.367 | 0.018 |  |
| Model 4: additionally adjusted for trunk fat mass and limb fat mass | –2.731(–4.789 to –0.674) | –0.195 | –2.610 | 0.009 | 2.4% |
| **Impacts of limb lean mass on AUC_glucagon_** |  |  |  |  |  |
| Model 0: crude; | –8.433(–13.04 to –3.824) | –0.174 | –3.597 | <0.001 |  |
| Model 1: adjusted for age, diabetes duration, BMI, SBP, DBP and statin medication; | –8.693(–15.25 to –2.134) | –0.180 | –2.605 | 0.010 |  |
| Model 2: additionally adjusted for ALT, albumin, lipid profiles, UA, eGFR and TBI; | –9.710(–16.44 to –2.978) | –0.201 | –2.836 | 0.005 |  |
| Model 3: additionally adjusted for HbA1c, ISI_C-peptide_, AUC_C-peptide_ and glucose-lowering therapies; | –8.349(–15.36 to –1.340) | –0.172 | –2.342 | 0.020 |  |
| Model 4: additionally adjusted for trunk fat mass and limb fat mass | –8.380(–15.61 to –1.155) | –0.172 | –2.281 | 0.023 | 1.5% |

ISI_C-peptide_: C-peptide-substituted Matsuda’s index; lnISI_C-peptide_: natural log-transformed ISI_C-peptide_; AUC_C-peptide_: C-peptide area under curve during OGTT; lnAUC_C-peptide_: natural log-transformed AUC_C-peptide_; AUC_glucagon_: glucagon area under the curve during OGTT; BMI: body mass index; ALT: alanine aminotransferase; TBI: total bilirubin; UA: uric acid; HbA1c: glycosylated hemoglobin A1c; UA: uric acid; eGFR: estimated glomerular filtration rate.
