# Supplementary material for: Connections between body composition and dysregulation of islet α- and β-cells in type 2 diabetes
Source: Diabetol Metab Syndr. 2024 Jan 9;16:11. doi: 10.1186/s13098-023-01250-3 (PMC10775650; doi:10.1186/s13098-023-01250-3)
Supplement: Supplementary file 7 — Additional file 7: Table S5. Impact of body composition on outcomes of islet α- and β-cell functions according to multivariate linear regression analysis in women with T2D (n = 315). [file 13098_2023_1250_MOESM7_ESM.docx]

**Table S5** Impact of body composition on outcomes of islet α- and β-cell functions according to multivariate linear regression analysis in women with T2D (n=315)

| **Models** | **B (95% CI)** | ***β*** | ***t*** | ***p*** | ***Partial R^2^*** |
| --- | --- | --- | --- | --- | --- |
| **Impacts of trunk fat mass on lnISI_C-peptide_** |  |  |  |  |  |
| Model 0: crude; | –0.045(–0.065 to –0.025) | –0.247 | –4.497 | <0.001 |  |
| Model 1: adjusted for age, diabetes duration, BMI, SBP, DBP and statin medication; | –0.037(–0.073 to –0.001) | –0.203 | –1.995 | 0.046 |  |
| Model 2: additionally adjusted for ALT, albumin, lipid profiles, UA, eGFR and TBI; | –0.036(–0.072 to –0.001) | –0.201 | –2.015 | 0.045 |  |
| Model 3: additionally adjusted for HbA1c, fasting glucagon, AUC_glucagon_ and glucose-lowering therapies | –0.037(–0.073 to –0.001) | –0.205 | –2.014 | 0.045 |  |
| Model 4: additionally adjusted for trunk lean mass and limb lean mass | –0.038(–0.074 to –0.001) | –0.207 | –2.021 | 0.044 | 6.0% |
| **Impacts of trunk fat mass on lnAUC_C-peptide_** |  |  |  |  |  |
| Model 0: crude; | 0.045(0.027 to 0.062) | 0.274 | 5.035 | 0.016 |  |
| Model 1: adjusted for age, diabetes duration, BMI, SBP, DBP and statin medication; | 0.040(0.007 to 0.072) | 0.244 | 2.426 | <0.001 |  |
| Model 2: additionally adjusted for ALT, albumin, lipid profiles, UA, eGFR and TBI; | 0.037(0.005 to 0.069) | 0.228 | 2.294 | 0.022 |  |
| Model 3: additionally adjusted for HbA1c, fasting glucagon, AUC_glucagon_ and glucose-lowering therapies | 0.037(0.006 to 0.067) | 0.226 | 2.376 | 0.018 |  |
| Model 4: additionally adjusted for trunk lean mass and limb lean mass | 0.040(0.009 to 0.070) | 0.245 | 2.580 | 0.010 | 5.6% |
| **Impacts of limb lean mass on fasting glucagon** |  |  |  |  |  |
| Model 0: crude; | –2.327(–4.211 to –0.443) | –0.136 | –2.430 | 0.016 |  |
| Model 1: adjusted for age, diabetes duration, BMI, SBP, DBP and statin medication; | –2.552(–5.176 to 0.072) | –0.149 | –1.914 | 0.057 |  |
| Model 2: additionally adjusted for ALT, albumin, lipid profiles, UA, eGFR and TBI; | –3.275(–5.978 to –0.572) | –0.191 | –2.385 | 0.018 |  |
| Model 3: additionally adjusted for HbA1c, ISI_C-peptide_, AUC_C-peptide_ and glucose-lowering therapies; | –3.779(–6.606 to –0.952) | –0.220 | –2.631 | 0.009 |  |
| Model 4: additionally adjusted for trunk fat mass and limb fat mass | –3.870(–6.772 to –1.017) | –0.226 | –2.670 | 0.008 | 2.4% |
| **Impacts of limb lean mass on AUC_glucagon_** |  |  |  |  |  |
| Model 0: crude; | –10.68(–17.91 to –3.445) | –0.162 | –2.905 | 0.004 |  |
| Model 1: adjusted for age, diabetes duration, BMI, SBP, DBP and statin medication; | –11.86(–21.77 to –1.950) | –0.180 | –2.355 | 0.019 |  |
| Model 2: additionally adjusted for ALT, albumin, lipid profiles, UA, eGFR and TBI; | –12.12(–22.39 to –1.846) | –0.184 | –2.322 | 0.021 |  |
| Model 3: additionally adjusted for HbA1c, ISI_C-peptide_, AUC_C-peptide_ and glucose-lowering therapies; | –11.48(–22.28 to –0.689) | –0.174 | –2.094 | 0.037 |  |
| Model 4: additionally adjusted for trunk fat mass and limb fat mass | –11.90(–22.78 to –1.009) | –0.180 | –2.151 | 0.032 | 1.9% |

ISI_C-peptide_: C-peptide-substituted Matsuda’s index; lnISI_C-peptide_: natural log-transformed ISI_C-peptide_; AUC_C-peptide_: C-peptide area under curve during OGTT; lnAUC_C-peptide_: natural log-transformed AUC_C-peptide_; AUC_glucagon_: glucagon area under the curve during OGTT; BMI: body mass index; ALT: alanine aminotransferase; TBI: total bilirubin; UA: uric acid; HbA1c: glycosylated hemoglobin A1c; UA: uric acid; eGFR: estimated glomerular filtration rate.
